# Artificial Intelligence in Differential Diagnostics of Meningitis: A Nationwide Study

**DOI:** 10.3390/diagnostics11040602

**Published:** 2021-03-28

**Authors:** Alexios-Fotios A. Mentis, Irene Garcia, Juan Jiménez, Maria Paparoupa, Athanasia Xirogianni, Anastasia Papandreou, Georgina Tzanakaki

**Affiliations:** 1 National Meningitis Reference Laboratory, Department of Public Health Policy, School of Public Health, University of West Attica, 122 43 Athens, Greece; axirogianni@uniwa.gr (A.X.); npapandreou@uniwa.gr (A.P.); gtzanakaki@uniwa.gr (G.T.); 2 Department of Mathematical Sciences and Informatics, and Health Research Institute (IdISBa), University of the Balearic Islands (UIB), 07122 Palma, Balearic Islands, Spain; irene.garcia@uib.es; 3 ADEMA University School, University of the Balearic Islands (UIB), 07122 Palma, Balearic Islands, Spain; j.jimenez@eua.edu.es; 4 Department of Intensive Care Medicine, University Medical Center Hamburg-Eppendorf, 20251 Hamburg, Germany; m.paparoupa@uke.de

**Keywords:** meningitis, bacterial infection, viral infection, neutrophil-to-lymphocyte ratio, artificial intelligence, machine learning

## Abstract

Differential diagnosis between bacterial and viral meningitis is crucial. In our study, to differentiate bacterial vs. viral meningitis, three machine learning (ML) algorithms (multiple logistic regression (MLR), random forest (RF), and naïve-Bayes (NB)) were applied for the two age groups (0–14 and >14 years) of patients with meningitis by both conventional (culture) and molecular (PCR) methods. Cerebrospinal fluid (CSF) neutrophils, CSF lymphocytes, neutrophil-to-lymphocyte ratio (NLR), blood albumin, blood C-reactive protein (CRP), glucose, blood soluble urokinase-type plasminogen activator receptor (suPAR), and CSF lymphocytes-to-blood CRP ratio (LCR) were used as predictors for the ML algorithms. The performance of the ML algorithms was evaluated through a cross-validation procedure, and optimal predictions of the type of meningitis were above 95% for viral and 78% for bacterial meningitis. Overall, MLR and RF yielded the best performance when using CSF neutrophils, CSF lymphocytes, NLR, albumin, glucose, gender, and CRP. Also, our results reconfirm the high diagnostic accuracy of NLR in the differential diagnosis between bacterial and viral meningitis.

## 1. Introduction

Mitigating meningitis remains both a global health challenge and a clinical emergency issue, the latter even in resource-rich settings [1,2]. Of paramount importance is its prompt diagnosis and, in particular, the differential diagnosis between the two main categories, bacterial and viral meningitis [3]. The latter is crucial for two main reasons: (a) failure to deliver proper antibiotic therapy in bacterial meningitis can lead to severe, permanent sequelae and invasive disease (especially due to *Neisseria meningitidis*) [4], and even death, and (b) unnecessary antibiotic or overtreatment of viral meningitis cases can lead to antimicrobial resistance, increased health care services cost, changes in human microbiome, and high levels of stress to the suffering patients [5]. Several differential diagnostic approaches have therefore been developed or proposed, ranging from simple procedures, such as measuring lactate and other parameters (for instance, albumino-cytological dissociation) [6,7], to sophisticated strategies, such as sequencing approaches, through metagenomics [8], host transcriptome analysis, or even single-cell RNA sequencing approaches [9,10,11].

We have previously investigated the promising role of cerebrospinal fluid (CSF) neutrophil-to-lymphocyte (NLR) ratio in the differential diagnosis of meningitis, both in the whole (i.e., all ages-inclusive) population [12] and at an age-specific scale [13]. NLR is a promising diagnostic biomarker because of *(a)* the high accuracy, especially for those aged over 14 years, and *(b)* the practicality and low cost, requiring only a CSF cell count analysis and a simple mathematical calculation [12,13]. Nonetheless, to our best knowledge, the combined effects with other CSF and blood parameters, beyond those referring to whole white cell counts, lymphocytes, and neutrophils, have not been explored in the differential diagnosis between bacterial and viral meningitis. Here, we attempted to address this hovering research gap by harnessing the power of artificial intelligence—notably, Machine Learning (ML)—approaches. In particular, we employed three different ML algorithms, and we found that the accuracy in the differential diagnosis of meningitis might be increased when these algorithms are used in a multivariate approach, instead of a ROC curve univariate treatment of the problem.

## 2. Materials and Methods

### 2.1. Patients, Setting, Laboratory Testing, and Diagnosis

A retrospective study of data from the Greek National Meningitis Reference Laboratory was performed, with the approach in regard to the diagnostic flow chart and the calculation of several biomarkers (e.g., blood soluble urokinase-type plasminogen activator receptor (suPAR), blood albumin, blood C-reactive protein (CRP) which is a protein related to the acute phase of inflammation, blood glucose) as previously described in the methodology [12,13,14]. Briefly, CSF samples were sent to the National Meningitis Reference Laboratory from adult and pediatric (that is, those hosting children aged less than 14 years) hospitals throughout the country. All samples were processed for diagnosis of bacterial meningitis mainly based on non-culture diagnosis with the application of two in-house, multiplex PCR techniques [14]; in particular, the latter consisted of a *single-tube PCR assay for the simultaneous detection of Neisseria meningitidis, Haemophilus influenzae type b and Streptococcus pneumoniae* [15] while, the second mPCR employed was for the simultaneous detection of *Haemophilus influenzae, Pseudomonas aeruginosa, Staphylococcus aureus, and Streptococcus spp.* [16] Calculations were performed as previously described using the age of 14-years-old as a binary classification [12,13] due to the age limit dividing referral to pediatric vs. adult hospitals in Greece.

### 2.2. Predicting the Type of Meningitis

After initial analysis, the data were divided into two groups (0–14 and >14 years) as previously applied [12,13]. Overall, the data consisted of 4339 cases (1758 and 2581 bacterial and viral meningitis, respectively) out of which there were 1737 viral and 940 bacterial meningitis cases among those aged 0–14 years. Οutliers using appropriate criteria were removed, and the definition of NLR used in previous studies [12,13] was retained. Furthermore, the CSF lymphocytes-to-blood-C-reactive protein ratio (LCR) by dividing the number of CSF lymphocytes with the values of CRP for each patient, following similar approaches in blood [17] was calculated. All data were processed using the free-access R language (https://cran.r-project.org/). The statistical significance level was set at <0.05.

Regarding the differential diagnosis based on ML algorithms, three machine learning (ML) algorithms, i.e., MLR, RF, and NB were applied for predicting the type of meningitis of patients in the three age groups (all the ages, 0–14 and >14 years) with meningitis diagnosed by both conventional (culture) and molecular (mPCR) methods. As a first step, the data set where the explanatory variables (or predictors) were CSF Neutrophils, CSF Lymphocytes, CSF NLR, Blood Albumin, Gender, Blood Glucose, Blood CRP, Blood suPAR, and LCR from these patients was defined, and the outcome was defined as the type of meningitis, respectively. During a second step, the dataset was split into two parts, i.e., the training set and the testing set, where (a) the training set was selected randomly as a set of cases from the data set, by fixing the number of training data points at 80% of the original data set, and (b) the testing set (20%) as the remaining data. Afterward, during a third step, a cross-validation procedure fitting each model was applied (likewise, it tuned the parameters of each model) using only part of the training set and examining how well the model predicted the testing set. The above indicated that the models were fitted to reproduce the outcomes corresponding to the predictor’s values from each patient in the training set; of note, after these parameters were tuned, the model was used to predict the outcomes corresponding to the predictor’s values for each patient in the testing set, and the percentage of right predictions (i.e., that coincides with the observed outcomes) were recorded. This procedure was repeated from the second step forward 500 times, and the mean values and 95% confidence intervals for these percentages were obtained from the resampled (bootstrapped) data. Specifically, the different sets of variables that were used as predictors from the number of available cases are shown in Table 1. In brief, Group 1 (G1) included the available values of the variables CSF Neutrophils and CSF Lymphocytes, Group 2 (G2) included the cases where there were available values of the variables from G1 and CSF NLR, Group 3 included the cases where values of the variables from G2 and Blood Albumin were available, Group 4 included the group of cases where values of the variables from G3 and Gender and Group were available, Group 5 included the cases of the variables included in G4 in addition to Blood Glucose, Group 6 included the group of cases from the G5 values in addition to Blood CRP, Group 7 included the values of the variables obtained from G6 in addition to values from Blood suPAR, and Group 8 included the group of cases where values of the variables from G7 and LCR were available.

## 3. Results

### 3.1. Differential Diagnosis of Meningitis

Three standard ML algorithms—multivariate logistic regression (MLR), random forest (RF), and naïve-Bayes (NB)—were applied. For the first two models—MLR and RF—the most important predictors were reportedly straightforward when using the R libraries. We noted that several sets of covariates leading to good performance were demonstrated when ML was used for predicting the type of meningitis (Table 2). Depicted in the following tables of note are: the analyses using CSF neutrophils and CSF lymphocytes as predictors (Table 2a); those using CSF neutrophils, CSF lymphocytes, and NLR as predictors (Table 2b); those using CSF neutrophils, CSF lymphocytes, NLR, and blood albumin as predictors (Table 2c); those using CSF neutrophils, CSF lymphocytes, NLR, albumin, glucose, age group, and gender as predictors (Table 2d); those using CSF neutrophils, CSF lymphocytes, NLR, blood albumin, glucose, age group, gender, and CRP as predictors (Table 2e); those using CSF neutrophils, CSF lymphocytes, NLR, blood albumin, blood glucose, age group, gender, CRP, and blood suPAR as predictors (Table 2f), and; those using CSF neutrophils, CSF lymphocytes, NLR, blood albumin, blood glucose, age group, gender, blood CRP, blood suPAR, and LCR as predictors (Table 2g).

#### 3.1.1. Group Aged 0–14 Years

For the study group of 0–14 years of age, the same cross-validation procedure as that used in the age-independent case when applying ML algorithms was applied. Using CSF neutrophils and CSF lymphocytes as predictors, we found 1051 available cases distributed among 672 (64%) viral and 379 (36%) bacterial cases (Table 3). Our training set used 840 cases, and our testing set had 211 cases (Table 3a). Using CSF neutrophils, CSF lymphocytes, and NLR as predictors, we identified 1044 available cases distributed in 670 (64%) viral and 374 (36%) bacterial cases with a training set of 835 cases, and a testing set of 209 cases (Table 3b). Using CSF neutrophils, CSF lymphocytes, NLR, and blood albumin as predictors, we identified 983 available cases distributed among 635 (65%) viral and 348 (35%) bacterial cases with a training set of 786 cases, and a testing set of 197 cases (Table 3c). Using CSF neutrophils, CSF lymphocytes, NLR, blood albumin, blood glucose, and gender as predictors, we identified 966 available cases distributed among 631 (65%) viral and 335 (35%) bacterial cases with a training set of 772 cases, and testing set of 194 cases (Table 3d). Using CSF neutrophils, CSF lymphocytes, NLR, blood albumin, blood glucose, gender, and blood CRP as predictors, there were 691 available cases distributed among 432 (63%) viral and 259 (37%) bacterial cases with a training set of 552 cases, and testing set of 139 cases (Table 3e).

Although the cases below have few data, an attempt to perform the calculations in question for the sake of completeness was made. In doing so, using CSF neutrophils, CSF lymphocytes, NLR, blood albumin, blood glucose, gender, blood CRP, and blood suPAR as predictors, we identified 99 available cases distributed among 59 (60%) viral and 40 (40%) bacterial cases with a training set of 79 cases, and testing set of 20 cases (Table 3f). Using CSF neutrophils, CSF lymphocytes, NLR, blood albumin, blood glucose, gender, blood CRP, blood suPAR, and LCR as predictors, we identified 99 available cases distributed among 59 (60%) viral and 40 (40%) bacterial cases (training set of 79 cases, and testing set of 20 cases; Table 3g).

#### 3.1.2. Age Group over 14 Years

In the group aged over 14 years, 824 viral (source = 1) and 803 bacterial (source = 2) meningitis cases (in total, 1662 cases) were identified. Outliers using appropriate criteria were removed, and the same cross-validation procedure when applying ML algorithms was applied (Table 4); by the use of CSF neutrophils and CSF lymphocytes as predictors, we identified 791 available cases distributed among 317 (40%) viral and 474 (60%) bacterial cases (Table 4a). By using the combination of CSF neutrophils, CSF lymphocytes, and NLR as covariate predictors, we identified 782 available cases distributed among 316 (40%) viral and 466 (60%) bacterial cases (Table 4b), by using the CSF neutrophils, CSF lymphocytes, NLR, and blood albumin as predictors, we identified 685 available cases distributed among 283 (41%) viral and 402 (59%) bacterial cases (Table 4c), by using CSF neutrophils, CSF lymphocytes, NLR, blood albumin, blood glucose, and gender as predictors, we identified 640 available cases distributed among 280 (44%) viral and 360 (56%) bacterial cases, (Table 4d), using CSF neutrophils, CSF lymphocytes, NLR, blood albumin, blood glucose, gender, and blood CRP as predictors, we identified 264 available cases distributed among 121 (46%) viral and 143 (54%) bacterial cases. Our training set had 211 cases and our testing set had 53 cases (Table 4e). Nonetheless, the combinations below represent only 26 available cases and are, therefore, too small to justify applying ML—that is, (a) using CSF neutrophils, CSF lymphocytes, NLR, blood albumin, blood glucose, gender, blood CRP, and blood suPAR as predictors, and (b) using CSF neutrophils, CSF lymphocytes, NLR, albumin, blood glucose, gender, blood CRP, blood suPAR, and LCR as predictors.

## 4. Discussion

**Figure 1:**
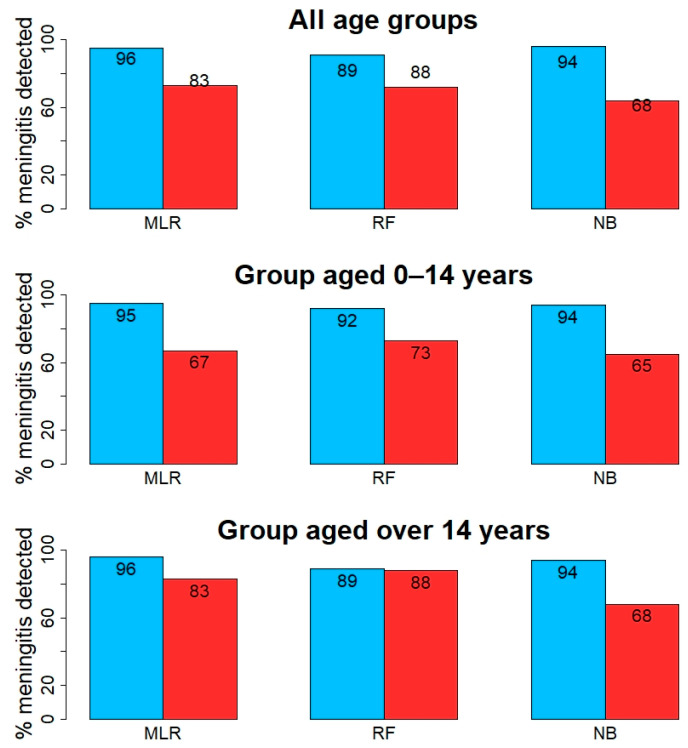
Summary plot whereby the optimal results (expressed as a percentage (%) of meningitis cases detected) are displayed for each Machine Learning (ML) model, i.e., Multiple logistic regression (MLR), Random forest (RF), and Naïve-Bayes (NB). Blue bars correspond to viral and red to bacterial meningitis.

Our study represents the first attempt on assessing the prognosis of meningitis based NLR and other covariates through the use of ML approaches (Figure 1), as those are gaining increasing interest in clinical microbiology [18] and, alongside clinical guidelines for AI implementation in clinical decisions, they are expected to reach a clinical prime time in the coming decade [19]. Most of the previous studies in the differential diagnosis between viral and bacterial meningitis involve a ROC-Area under the curve (AUC)-like analysis, where only one variable was considered at one time. For instance, sensitivity and specificity should be computed for different threshold values of NLR alone, and then the ROC generated for finally getting the AUC for NLR, at which the analysis is individually performed for the AUC of the other covariates. This prevents the identification of synergistic combinations of the variables and their use for optimized predictions. On the contrary, when applying an ML-based procedure, a set of variables is considered together (CSF neutrophils, NLR, albumin, and so on) in order to generate the predicted value of the response (that is, the type of meningitis), and then this prediction is compared with the observed value to compute the percentage of viral cases being correctly predicted and the percentage of bacterial cases being correctly predicted.

From an initial set of predictors that included only CSF neutrophils and CSF lymphocytes, the present study increased the number of predictors one by one in steps by the implementation of a cross-validation procedure at each step, starting by training the ML models (that is, tuning the parameters) on a randomly selected subset of cases, and further using the fitted models to predict the type of meningitis on a new *“not seen before”* dataset (testing set) and computed the percentages of correct predictions, by repeating this procedure 500 times and computed 95% confidence intervals from the predictions.

Although the followed forward stepwise scheme gave a limited combination of predictors, our results demonstrated the important role of NLR when it is used as a predictor in ML algorithms for the differential diagnosis between viral and bacterial meningitis, building upon previous studies on NLR’s validity in different diseases, even chronic ones such as cancer [20]. Furthermore, our results highlight the emerging applications of ML approaches in medicine, in a whole spectrum from diabetes and cancer to infectious diseases and sepsis-associated events [21,22,23]. Of note, regarding the prediction of diseases in general, the most to least frequently applied algorithms are the support vector machine, NB, and RF algorithms, respectively; nonetheless, the latter algorithm presented the highest accuracy in a previous study that aimed to compare the above algorithms [24], a finding also corroborated in recent studies in young febrile patients [25].

This study’s limitations include the lack of validation on meningitis epidemics-afflicted resource-poor settings, and the possibility that some patients suspected of meningitis could have meningitis due to *M. tuberculosis* and HIV-related or other central nervous system infections; both types relatively rare in Greece. Moreover, some outliers that were removed may likely be encountered in daily clinical practice; however, only a very small number of cases have been removed which is unlikely to affect the outcomes of this study.

As this is the first attempt on assessing the prognosis of meningitis based on our covariates, future multicenter studies in the field are needed for further individualized predictions—for instance, as part of meningitis prognostic scores during the P4 (Predictive, Preventive, Personalized and Participatory) Medicine era [26,27]. Moreover, regarding linking clinical data with CSF neutrophil counts and NLR, future studies could link all the clinical phenotypes with CSF and NLR, to potentially identify other biomarkers that are crucial for specific clinical phenotypes, as previously described [13]. Lastly, future studies could assess what are the cellular and signalling underpinnings explaining why NLR is of additive value to neutrophils and lymphocytes in the differential diagnosis of meningitis, e.g., following studies on neutrophils-related signatures [28].

## 5. Conclusions

In conclusion, from the results of the percentages of the rightly predicted type of meningitis in Table 2, Table 3 and Table 4, it can be concluded that ML models may be used as an accurate method to predict whether a patient has viral or bacterial meningitis from their values for CSF neutrophils, CSF lymphocytes, NLR, albumin, glucose, gender, and CRP and might be included in the mainstream of computer-aided diagnosis systems for this purpose. Of note, for both age groups, the MLR model consistently predicted the percentage of viral meningitis more accurately than the other two models, RF was the best model when predicting bacterial meningitis, whereas NB showed the lowest performance. This indicates that a combination of these two models could potentially optimize the differential diagnosis.

## 6. Patents

No patent applicable.

## Figures and Tables

**Table 1:** Displayed number of available cases for the different sets of explanatory variables based on 4339 meningitis cases.

Covariates	Total Number of Cases	Viral Cases	Bacterial Cases
G1 = CSF Neutrophils + CSF Lymphocytes	1860	1005 (54%)	855 (46%)
G2 = G1 + CSF NLR	1844	1002 (54%)	842 (46%)
G3 = G2 + Blood Albumin	1684	932 (55%)	752 (45%)
G4 = G3 + Gender + Group	1668	918 (55%)	750 (45%)
G5 = G4 + Blood Glucose	1606	911 (57%)	695 (43%)
G6 = G5 + Blood CRP	955	553 (58%)	402 (42%)
G7 = G6 + Blood suPAR	125	69 (55%)	56 (45%)
G8 = G7 + LCR	125	69 (55%)	56 (45%)

**Table 2:** Differential diagnosis of meningitis based on machine learning algorithms using different combinations of covariates across all age groups.

ML Algorithm	Percentage of Viral Meningitis Detected: Mean Value and CI (95%)	Percentage of Bacterial Meningitis Detected: Mean Value and CI (95%)	Most Important Predictor
**Table 2a**			
MLR	96% (92%, 99%)	49% (40%, 58%)	CSF Neutrophils
RF	86% (79%, 90%)	61% (51%, 70%)	CSF Neutrophils
NB	96% (94%, 99%)	44% (35%, 54%)	NA
**Table 2b**			
MLR	95% (91%, 97%)	68% (61%, 75%)	NLR
RF	87% (81%, 90%)	78% (70%, 82%)	CSF Neutrophils
NB	96% (92%, 98%)	60% (50%, 68%)	NA
**Table 2c**			
MLR	96% (93%, 99%)	66% (58%, 74%)	NLR
RF	91% (86%, 65%)	72% (65%, 79%)	CSF Neutrophils
NB	95% (91%, 98%)	63% (53%, 71%)	NA
**Table 2d**			
MLR	95% (92%, 98%)	73% (66%, 79%)	NLR
RF	90% (85%, 93%)	71% (64%, 79%)	CSF Neutrophils
NB	96% (93%, 99%)	64% (56%, 73%)	NA
**Table 2e**			
MLR	95% (92%, 98%)	73% (66%, 79%)	NLR
RF	90% (85%, 93%)	78% (72%, 84%)	CSF Neutrophils
NB	95% (92%, 98%)	66% (59%, 74%)	NA
**Table 2f**			
MLR	95% (90%, 99%)	62% (50%, 74%)	NLR
RF	91% (86%, 95%)	76% (70%, 83%)	NLR
NB	96% (92%, 100%)	52% (38%, 66%)	NA
**Table 2g**			
MLR	86% (63%, 100%)	72% (43%, 100%)	NLR
RF	93% (87%, 97%)	69% (57%, 81%)	NLR
NB	88% (67%, 100%)	64% (22%, 89%)	NA

**Table 3:** Differential diagnosis of meningitis based on machine learning algorithms using different combinations of covariates in those aged 0–14 years.

ML Algorithm	Percentage of Viral Meningitis Detected: Mean Value and CI (95%)	Percentage of Bacterial Meningitis Detected: Mean Value and CI (95%)	Most important predictor
**Table 3a**			
MLR	97% (94%, 99%)	56% (46%, 66%)	CSF Neutrophils
RF	89% (83%, 94%)	67% (56%, 76%)	CSF Neutrophils
NB	96% (93%, 99%)	42% (32%, 52%)	NA
**Table 3b**			
MLR	96% (93% 99%)	61% (50%, 72%)	NLR
RF	89% (83%, 94%)	66% (55%, 76%)	CSF Neutrophils
NB	95% (92%, 98%)	55% (44%, 65%)	NA
**Table 3c**			
MLR	96% (93%, 99%)	63% (53%, 74%)	NLR
RF	90% (85%, 95%)	67% (57%, 77%)	CSF Neutrophils
NB	95% (91%, 98%)	60% (50%, 71%)	NA
**Table 3d**			
MLR	95% (90%, 98%)	63% (53%, 74%)	CSF Neutrophils
RF	90% (85%, 95%)	67% (57%, 77%)	CSF Neutrophils
NB	95% (91%, 98%)	60% (50%, 71%)	NA
**Table 3e**			
MLR	95% (91%, 99%)	67% (56%, 78%)	NLR
RF	90% (83%, 96%)	70% (58%, 81%)	CSF Neutrophils
NB	94% (89%, 99%)	65% (53%, 77%)	NA
**Table 3f**			
MLR	89%% (73%, 100%)	67% (33%, 100%)	NLR
RF	84%% (58%, 100%)	69%% (33%, 97%)	NLR
NB	86% (64%, 97%)	61% (29%, 97%)	NA
**Table 3g**			
MLR	86% (64%, 100%)	61% (29%, 97%)	NLR
RF	92% (77%, 100%)	73% (41%, 97%)	NLR
NB	85%% (61%, 100%)	66% (33%, 97%)	NA

NLR refers to Neutrophil-to-Lymphocyte Ratio. MLR refers to multiple logistic regression. RF refers to random forest. NB refers to naïve-Bayes. NA refers to non-available.

**Table 4:** Differential diagnosis of meningitis based on machine learning algorithms using different combinations of covariates in those aged over 14 years.

ML Algorithm	Percentage of Viral Meningitis Detected: Mean Value and CI (95%)	Percentage of Bacterial Meningitis Detected: Mean Value and CI (95%)	Most Important Predictor
**Table 4a**			
MLR	97% (94%, 100%)	75% (67%, 82%)	CSF Neutrophils
RF	82% (71%, 91%)	86% (79%, 92%)	CSF Neutrophils
NB	97% (91%, 100%)	51% (40%, 65%)	NA
**Table 4b**			
MLR	96% (91%, 100%)	83% (75%, 90%)	NLR
RF	84% (75%, 92%)	85% (78%, 91%)	CSF Neutrophils
NB	96% (90%, 100%)	65% (53%, 79%)	NA
**Table 4c**			
MLR	95% (89%, 100%)	83% (75%, 90%)	NLR
RF	87% (78%, 95%)	87% (80%, 93%)	CSF Neutrophils
NB	94% (89%, 98%)	68% (58%, 81%)	NA
**Table 4d**			
MLR	95% (89%, 100%)	81% (74%, 89%)	CSF Neutrophils
RF	89% (80%, 96%)	88% (80%, 94%)	CSF Neutrophils
NB	94% (88%, 100%)	65% (51%, 79%)	NA
**Table 4e**			
MLR	94% (84%, 100%)	82% (70%, 93%)	NLR
RF	89% (76%, 100%)	87% (73%, 97%)	CSF Neutrophils
NB	95% (84%, 100%)	59% (37%, 82%)	NA

## Data Availability

The data presented in this study are available on reasonable request from the corresponding author on the condition of relevant approval from the originating Institution.
